# Plasmacytoma/multiple myeloma type posttransplant lymphoproliferative disorder

**DOI:** 10.1002/jha2.386

**Published:** 2022-01-24

**Authors:** Theodora A.M. Claushuis, Lidwine W. Tick, Peter van Zwam

**Affiliations:** ^1^ Hematology Maxima Medisch Centrum Eindhoven The Netherlands


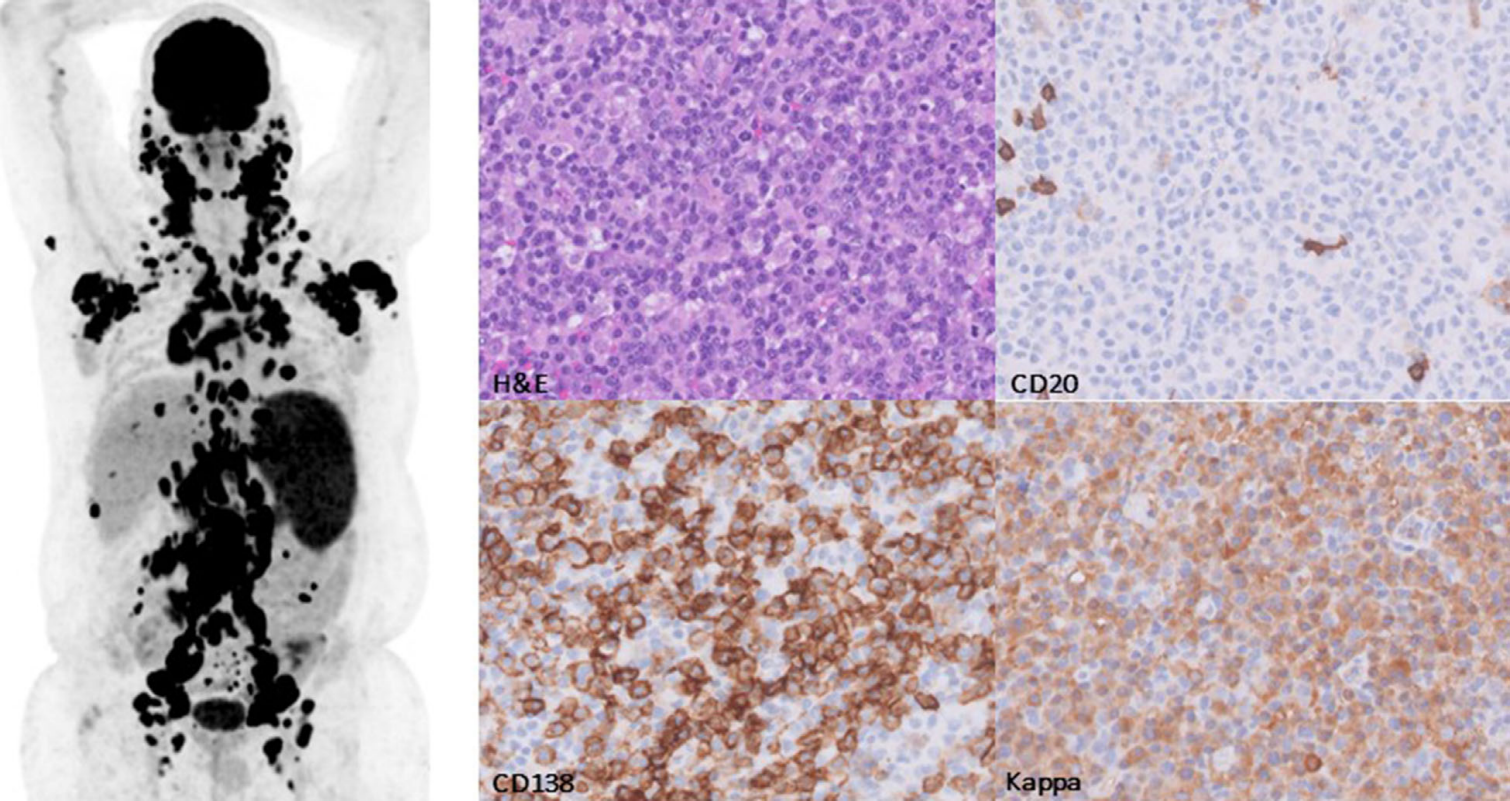


A 38‐year‐old male with a history of lupus nephritis and kidney transplantation 3 years ago, presented with fever, night sweats, and lymphadenopathy while being treated with tacrolimus, mycophenolate mofetil, and prednisone. Evaluation with PET and CT scan showed generalized lymphadenopathy and splenomegaly. (Left‐sided figure). Biopsy of an inguinal lymph node showed a monotypic plasma cell proliferation (CD138+, kappa+, CD20‐), as part of a monomorphic variant of post‐transplant lymphoproliferative disease (PTLD) (Right‐sided figure). The specific type was determined as plasmacytoma/multiple myeloma type PTLD. However, a diagnosis of PTLD type plasma cell‐rich marginal zone lymphoma was also possible, although no separate malignant lymphocytic B‐cell component could be demonstrated. No Epstein–Barr virus (EBV) positivity was found. Bone‐marrow showed a similar monomorphic PTLD with an increased amount of plasma cells. Laboratory testing showed only mild anemia and no kidney function decline or hypercalcemia. M‐protein was identified as IgA‐kappa, 24 g/L. There were no bone lesions. Cytogenetics of the bone marrow showed no abnormalities. A diagnosis of plasmacytoma/multiple myeloma type PTLD was made, and treatment was commenced with thalidomide, bortezomib, and dexamethasone.

Multiple myeloma/plasmacytoma‐like PTLD is a rare complication of solid organ transplant. It is characterized by plasmacytic histology with the presence of CD138 and lack of CD20. Only 3.5% of PTLD are of this subtype [1]. In a retrospective cohort and case series, the median time from organ transplant to disease was 3–10 years [1, 2, 3, 4]. Bone lesions were only present in a minority of patients [2] and many patients presented with extranodal disease [1, 3, 4] Similar to our patient, no significant hypercalcemia or renal insufficiency was found, and only mild anemia [3, 4] Patients had both positive and negative EBV immunohistochemical staining [2, 3, 4]. Patients underwent a reduction in immunosuppression and treatment with proteasome inhibitor‐based regimens with 20% also receiving autologous stem cell transplants. The median overall survival was 2.4 years and survival was decreased compared to regular multiple myeloma patients [1].

In conclusion, plasmacytoma/multiple myeloma type PTLD often presents without bone lesions, hypercalcemia, or renal insufficiency. Differentiation between this entity and other PTLD's with a prominent component of plasma cells (including marginal zone lymphoma) can be difficult, as both can produce monoclonal paraproteins. Treatment for plasmacytoma/multiple myeloma type PTLD is currently similar to conventional myeloma, however outcomes are inferior.

## References

[1] Rosenberg AS , Ruthazer R , Paulus JK , Kent DM , Evens AM , Klein AK . Survival analyses and prognosis of plasma cell myeloma and plasmacytoma‐like post‐transplant lymphoproliferative disorders. Clin Lymphoma Myeloma Leuk. 2016;16(12):684–92 27771291 10.1016/j.clml.2016.09.002 PMC5402751

[2] Ofori K , Soderquist CR , Murty VV , Park D , Vlad G , Leeman‐Neill RJ , et al. The clinical and pathological features of plasma cell myeloma post solid organ transplantation. Am J Hematol. 2020;95:1531–41. 32864761 10.1002/ajh.25988

[3] Sun X , Peterson LoAC , Gong Y , Traynor AE , Nelson BP . Post‐transplant plasma cell myeloma and polymorphic lymphoproliferative disorder with monoclonal serum protein occurring in solid organ transplant recipients. Mod Pathol. 2004;17(4):389–94 14976525 10.1038/modpathol.3800080

[4] Karuturi M , Shah N , Frank D , Fasan O , Reshef R , Ahya VN , et al. Plasmacytic post‐transplant lymphoproliferative disorder: a case series of nine patients. Transpl Int. 2013;26(6):616–22 23551167 10.1111/tri.12091

